# Exploring the Needs of Stakeholders For Successful Patient Involvement in Mental Health Education

**DOI:** 10.3389/phrs.2025.1608124

**Published:** 2025-03-12

**Authors:** Marlin Klarenbeek, Eduard de Bruin, Yudit Namer

**Affiliations:** Department of Psychology, Health and Technology, University of Twente, Enschede, Netherlands

**Keywords:** patient involvement, mental health education, scoping reviews, checklist, content analysis

## Abstract

**Objective:**

This scoping review aims to comprehensively map the existing literature on Patient Involvement (PI) in mental health education (MHE), identify the needs of mental health (MH) educators, students, and patients with lived experiences of MH challenges and develop a checklist for successful implementation of PI in MHE.

**Methods:**

Conducted between November 2023–January 2024, this review followed PRISMA-ScR guidelines in databases PubMed, Scopus, ProQuest Dissertations and Theses, and WHO. Eligibility criteria adhered to PICOS guidelines, and screening was done via Covidence. Content analysis was carried out to develop a checklist.

**Results:**

Eleven qualitative articles were found, revealing two superordinate stakeholder needs categories: Interpersonal and Course Needs. Interpersonal Needs included Self-determination, Communication and Collaboration, Recognition and Support, and Holistic approach. Course Needs comprised Content, Organisational, and Teaching. A checklist was developed to support PI in MHE.

**Conclusion:**

Guidelines for successful PI in MHE should prioritize patient autonomy, foster collaboration, provide support, ensure inclusive course content, and promote patient involvement in educational processes. Study limitations, such as potential bias, underscore the need for future research to enhance evidence-based practices in MHE.

## Introduction

Mental health education (MHE) is evolving to integrate the perspectives of individuals with firsthand mental health service (MHS) experience. This signifies a shift from a traditional passive role towards an active involvement of patients in students’ clinical education. In consonance with the findings of Simmons et al. and Costa et al. about individuals receiving mental health treatment, the word patient is used consistently throughout this review report and when an original article occupies a different name to indicate patients [1, 2]. This approach of Patient Involvement (PI) in MHE views mental health patients as *experts by experience*, (EBE) who contribute positively to students’ and EBE’s attitudes and wellbeing [3–5]. PI has historically received more attention in physical health education than in MHE [6]. In clinical and communication skills teaching, PI began in the late 1970s with role-play patients, evolving into symptomatic PI programs by the 1980s by Stillman to enhance medical student examination [7]. PI disappeared from clinical education but resurged with the shift to the biopsychosocial model, emphasising patient expertise and collaboration [8, 9].

According to the prevailing model of PI of Towle and colleagues, three guiding principles shape patient inclusion in teaching [9]. First, the knowledge patients possess by experience cannot be generated by faculty. Second, for authentic inclusion of patients, power relations should be balanced. Third, patients should have a say in how and what to teach. Towle et al. also propose a hierarchical structure or taxonomy of PI [9]. The taxonomy ranges from 1 (low participation) to 6 (high participation): wherein 1) the patient is studied as a case; 2) the patient volunteers in clinical tutorials; 3) the patient shares experiences within a pre-established module framework; 4) the patient is actively teaching and evaluating students; 5) the patient contributes to curriculum development; 6) the patient has sustainable membership within the educational institute. When patients do take an active role within the curriculum of MHE, patients mostly take on the role of teacher, taking up a lower-to intermediate-level of involvement [10].

From the health professionals’ perspective, barriers to more active PI in MHE include the view that patients may not be fit for collaboration in decision-making (often in the case of severe mental illness), a lack of conceptual clarity of PI, increasing demands on health professionals’ time, and the biomedical model’s tendency to focus on patient’s limitations rather than strengths [11]. Despite these perceived barriers, professionals from multiple MH fields are increasingly willing to involve PI in MHE programmes [4].

PI in healthcare education has different meanings depending on the stakeholder’s perspective. For this reason, an assessment of the benefits and shortcomings of PI within healthcare education is lacking [11]. This knowledge gap leaves MHE curriculum developers lacking predefined guidelines for implementing PI and, with this, limiting the potentially positive effects of PI by minimising its implementation in educational programmes [4, 11]. This also reflects the lack of consensus of the definition of PI in MHE [12].

While policymakers and MHS administrators may also have important roles to play in PI implementation, stakeholders such as students, educators, and patients are directly involved in the MHE process and have a profound impact on its outcomes. Identifying their needs is crucial for tailoring educational approaches to address specific challenges or enhance positive aspects of PI in MHE [13]. This information is needed to lay the groundwork for developing an evidence-based fundament for PI implementation within MHE and identify areas where understanding and utilising PI is still underexplored. This fundament can be used by curriculum developers, educators and policymakers to establish guidelines for successful PI implementation in MHE programmes. In short, this study aims to a) comprehensively map the existing literature on PI in MHE, b) systematically analyse and identify the needs of the three main stakeholders of PI in MHE, and c) develop a checklist based on these identified needs.

The following review question has been formulated using the PICOS framework to reach the previously mentioned benefits of conducting a scoping review [14].

“What are the needs of students, mental health professionals and patients in PI in MHE?.”

## Methods

Scoping review was selected as the method due to the broad scope of the research topic and to highlight gaps in the existing literature to encourage future research in this dynamic and growing interdisciplinary field [15]. The scoping review protocol was based on established guidelines ([16], further refined by 15) and adheres to the PRISMA Extension for Scoping Reviews (PRISMA-ScR) [17]. This methodological framework and the prescribed reporting guideline aim to heighten the scoping review process’s replicability, and overall quality.

The literature research for this scoping review was conducted from November 2023 to January 2024. For this literature review, PubMed/MEDLINE, Scopus, ProQuest Dissertations and Theses, and World Health Organization databases were systematically searched to ensure a comprehensive perspective on the literature. Both grey literature and peer-reviewed sources were explored to enhance data comprehensiveness, capturing a broader spectrum of specific PI-related experiences. This provides a more holistic view of PI, which aligns with the study’s goals [18]. The ProQuest Dissertations and Theses and World Health Organization search engines were used to identify literature sources not findable in scientific databases for this purpose.

The eligibility criteria in Table 1 were used to screen articles included in this scoping review. The screening process consisted of 2 phases: 1) title and abstract screening and 2) full-text screening, both undertaken by MK in consultation with YN. Criteria refinement was an iterative process informed by preliminary literature searches, with ongoing adjustments based on ambiguities during screening. Both review phases were conducted using the Covidence screening software [19]. A standardised data extraction form based on the PRISMA-ScR guidelines was used to capture the concepts of interest within each article [20]. Reference snowballing, using both forward and backward searches, was employed to extend the database searches.

**TABLE 1:** Eligibility criteria (Exploring the Needs of Stakeholders for Successful Patient Involvement in Mental Health Education, Netherlands, 2024).

Aspect	Inclusion criteria	Exclusion criteria
Population	Students, educators and patients who uptake an active role in MHE	Patients are not actively involved in education (level 1 or 2 in Towle et al. [9]’s taxonomy
Context and scope	PI in MHE is addressed	General healthcare education or pharmacy education
Outcomes	Focus on the needs of stakeholders concerning PI: exploration of practical implications and experiences of PI in MHE	No mention of implicit or explicit needs of students, patients or educators
Types of Evidence	Qualitative and quantitative studies, mixed-methods research, systematic reviews, grey literature, dissertations, conference abstracts/proceedings, case studies, study protocols, and professional organization reports	None

**FIGURE 1:**
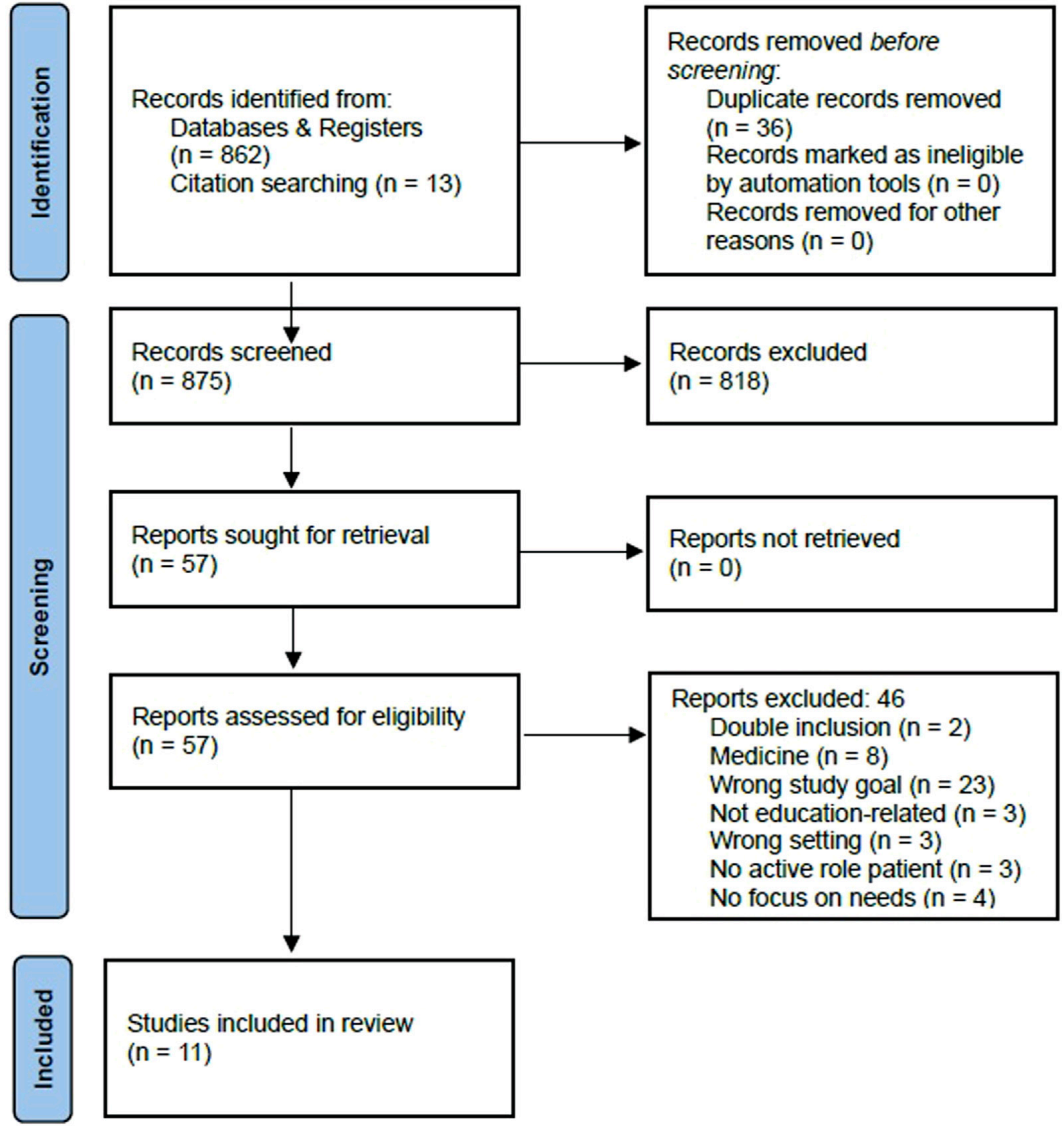
PRISMA flowchart of study selection (Exploring the Needs of Stakeholders for Successful Patient Involvement in Mental Health Education, Netherlands, 2024).

A pilot literature search by MK in PubMed established the initial keywords which were refined by YN. The search strategy (Table 2) included general search terms and specific search strings tailored for each database. PI in MHE encompasses various terms used for similar concepts, such as “patient involvement,” “consumer involvement,” and “service user involvement,” among others. Similarly, MHE spans diverse domains including clinical psychology, therapist training, and psychiatric nursing education. By formulating a search string that incorporates these terms and their synonyms, inclusivity and breadth is achieved. There were no predetermined restrictions on the studies’ publication date and geographic location, ensuring a thorough exploration of historical and contemporary literature. The study selection process is depicted in Figure 1, in which the number of excluded articles per step and the reason for exclusion are mentioned. The remaining articles were imported into the online study screening software Covidence [19].

**TABLE 2:** Search string (Exploring the Needs of Stakeholders for Successful Patient Involvement in Mental Health Education, Netherlands, 2024).

Key words and boolean operators
(“patient involvement” OR “consumer involvement” OR “service user involvement” OR “expert* by experience” OR “lived experience” OR “mental health patient*” OR “mental health service user*” OR “mental health survivor*” OR “mental health consumer*” OR “psychiatric survivor*” OR “client involvement” OR “patient engagement” OR “EBE”) AND (“mental health education” OR “clinical psychology program*” OR “clinical psychology training” OR “clinical psychology curriculum” OR “psychology education” OR “psychology training” OR “psychology program*” OR “psychology curricul*” OR “psychology learning” OR “psychology teaching” OR “psychology student* perspective*” OR “therapist training” OR “therapist education” OR “therapist program*” OR “therapist curricul*” OR “Mental Health Training” OR “Psychology Education” OR “Mental Health Counseling” OR “Psychiatric Nursing Education” OR “Mental Health Professional Training” OR “Therapist Education” OR “Counseling Psychology Training” OR “Psychotherapy Training” OR “Community Mental Health Training” OR “Recovery-Oriented Training” OR “Mental Health Worker Training”) AND (“need*” OR “requirement*” OR “expectation*” OR “desire*” OR “necessit*”)

The data charting form was developed within Covidence. Here, the study goal, the specific stakeholder that the article focuses on, the level of patient involvement (according to the model of Towle et al. [9]) and the identified needs for successful PI in MHE by the stakeholder education, were extracted from the articles. These categories were chosen to facilitate the comparison of data relevant for answering the research question. Data were gathered by reading the methods, results, and discussion sections of the 11 selected studies. The data extraction table is displayed in Table 3.

**TABLE 3:** Data extraction table (Exploring the Needs of Stakeholders for Successful Patient Involvement in Mental Health Education, Netherlands, 2024).

Study ID	Participant Info	Study objective	Stake-holder	Level of PI	Needs of investigated stakeholder(s)
Campbell and Wilson [21]	5 patients selected for participation Professional educators with >15 years of mental health service experience Ireland	To explore the experiences of service users participating in a clinical psychology training course. The aim was to address limitations identified in previous research and supplement existing knowledge by employing IPA to understand the psychological processes involved in such initiatives	Patients	2,3,4	To be acknowledged as colleagues rather than subjects of study Educators and students value lived experiences over academic knowledge in contributing to the training of MH professionals Having power and influence Use of forum to express needs and views in a respectful atmosphere Transformation, both of self and the MH systems
Clarke et al. [22]	10 randomly selected educators United Kingdom	Review benefits and barriers to PI	Educators	Various active levels	Genuine and integral involvement rather than tokenistic approaches Importance of strategic involvement at the management level for effective change Integration of PI with other aspects of course Equality between stakeholders; colleague educators are open to new experiences Enough personal, financial resources and less bureaucracy in Universities. Enhance accessibility of course for individuals outside the MH field
Happell et al. [23]	Educator group (n = 34) Patient group (n = 12) Australia	Present the perspectives and experiences of nurse academics and consumer educators regarding the feasibility and support for consumer participation roles in education for MH nursing	Educators and patients	Various active levels to ensure diversity	Reliability Need for organised and reliable patients Equality to general educational staff Vulnerability Recognise the burden on consumer and wellbeing issues and reduce tokenistic approach Patients that can handle vulnerability Support Systemic support and structured emotional support Seen as Griping Positive orientations are needed to gain a broader view Realistic and positive patients but not only those with ideas corresponding with views of academics
Happell et al. [24]	51 students, 43 female, 8 male Finland, Australia, Ireland, Iceland, Netherlands, Norway	To gather and present student feedback regarding their experiences with Experts by Experience (EBE) in the context of mental health education	Students	3,4	More incorporated EBE-led sessions to enhance understanding of mental distress. Correct planning within schedule to maximise benefits of the programme Greater consistency between patient content and assessments More structure within the course programme Include multiple perspectives. More balanced presentation of positive and negative experiences from patients
Happell et al. [25]	14 patients Finland, Australia, Ireland, Iceland, Netherlands, Norway	To examine the experience of being an EBE from the perspective of EBEs involved in the design, development and delivery of an EBE-led mental health nursing module	Patients	4,5	Collaboration with and support from nursing educators Autonomy in presenting patient perspective and presenting views inconsistent with those of academics. Emotional and practical support by formal and informal support mechanisms, team meetings, help in navigating (digital) systems and open-door policies Establish and maintain boundaries in sharing personal stories. Be able to consider purpose of the narrative and be selective about the details shared Adapt to needs of individual students at different educational stages
Horgan et al. [37]	50 patients participating in focus groups Iceland, Ireland, Finland, Netherlands, Norway and Australia	To develop a learning module based on service users’ perspectives, experiences and opinions about service user involvement in mental health nursing education	Patients	Involved in teaching (n = 3) not further defined	Support; including external supervision, teamwork, and debriefing. Support for students who find working with patients distressing Facilitate emotional, communicational and personal developments of students by course. Practical content to aid holistic understanding of mental illness More than one patient involved in teaching: diverse range of experiences. Stories that balance positive and negative aspects of experience Longer periods of involvement spread over the years for deeper engagement and reflection
Kang et al. [26]	98 psychiatry students at Semyung university, South Korea	To understand the impact of consumer involvement on nursing students’ attitudes towards mental health, their reflections on life, the learning experiences gained, and the preparation it provides for their future nursing careers	Students	3	Relating to lived experience to integrate theoretical knowledge and apply it More sessions with multiple patients, extended sessions and group discussions to improve own understanding More info about hospitalised consumers, more focus on nursing education and interaction with clinical experts in practice also to be integrated into the course
Kerry and Collett [27]	patient group of 10 + Group of 19 first-year trainee clinical psychologists in doctorate programme completing a survey United Kingdom	To examine the level and impact of EbE involvement in teaching on a UK DClinPsych course. It sought to gather information to generate change ideas and recommendations for EbE involvement in teaching	Patients and students	3, 4	Patients Further training in teaching and utilising online platforms Emotional support in boundary-keeping and handling distressing challenges to feeling empowered in teaching Bring about changes in themselves, students and future clients. Bringing purpose to lived experiences. Students Relating to intimidating lived experiences to gain confidence More informal contact with patients, and opportunities to ask questions Balance of positive and negative presented experiences, hearing different views Early exposure to emotional content to optimise skills and knowledge Emotional support for distressing patient content. Time to reflect on presented experiences Hearing wide range of perspectives, more diversity in the patient population and presented experience
Lea et al. [28]	Focus group of 8 patients (4 men, 4 women) 5 clinical psychology trainees (4 women, 1 mam) 5 members of course staff (5 women) United Kingdom	To elicit service users’, students’ and staff’s perceptions of the objectives and potential outcomes of service user involvement in clinical psychology training, in order to inform future questionnaire development	Patients, Educators, Students	3	Stay human through emotional intelligence development in educating students to ensure respectful future practice with cultural sensitivity Compassion for lived experience and emotional understanding, valuing lived experience more than academic knowledge Safe spaces to learn, learn from dealing with emotional experience and relating to it Learn how to instil meaning in life and self-determination; holistic approach (positive psychology approach). Reduction of the “them-us” divide Active role for patients in selection of the right patient for involvement Avoiding use of jargon
Meehan and Glover [29]	95 patients (mean age = 47.2, 54% women) involved in teaching in the London medical school psychiatry course	Explore the experiences of former MH consumers who have participated in the education and training of MH staff and students	Patients	4	Prevention from feeling exposed by presenting lived experience; not being asked inappropriate questions or seen as practice doll for diagnosing Educators needs to value contribution of patients; prevention of tokenistic approach Patients want to know what is expected from them in educational programmes
Walters et al. [30]	20 patients 12 educators 14 students All demographical diverse United Kingdom	To evaluate the impact of patient involvement in undergraduate medical education, specifically in the context of teaching medical students in community settings, with a focus on patients with common mental disorders	Patients, Educators, Students	3	Students show sympathy for patient Patients need distress relief by debriefing and prevention of intrusive questions by students and educators Patients need solid boundaries to decrease the experienced increased obligation

To analyse the data, content analysis was employed to identify patterns and themes, as well as systematically categorise and synthesise the data [31]. Content analysis is particularly suitable for the exploratory nature of a scoping review, providing a foundational understanding of the topic, accommodating and conceptually mapping the diverse data available in the included studies [32]. The coding process, conducted in ATLAS.ti 23.4.0 for Windows, involved iterative refinement as more data were analysed. A trustworthiness checklist was followed to ensure analytical rigour [33].

Data analysis was conducted inductively to identify essential concepts and facilitate analysis and mapping [34]. In the preparation phase, the results sections of the 11 selected articles underwent probability sampling due to the large volume of text with low relevance to the analysis theme [34]. In the organisation phase, the coding process started with open coding to create categories and abstract the data. Then, axial coding was used to group similar concepts into new themes. Finally, selective coding was used to adjust and finalise the coding scheme, forming a general description of the main contents within the articles [35]. The analysis aimed to achieve thematic saturation, with further analysis unlikely to yield substantial new information or significantly alter identified content themes [36]. Coding guidelines were established to extract different stakeholders’ (patients, educators and students) implicit and explicit needs from the articles, enhancing research transparency and trustworthiness, as well as facilitating systematic data exploration (Table 4).

**TABLE 4:** Guidelines for extracting needs from articles (Exploring the Needs of Stakeholders for Successful Patient Involvement in Mental Health Education, Netherlands, 2024).

Type of expressed need	Description of need	Example
Explicit Needs	Direct statements expressing needs. These are often straightforward and leave little room for interpretation	“A more equal relationship, not them thinking they are above you” [28]
Implicit Needs	Implicit needs may be implied or hinted at in the text. You may need to read between the lines to infer the needs of participants	“I’m waiting to see, that as I say our good faith in terms of how we’re involved and how we’re contributing is recognised.” [21].
Descriptive Language	Indicates dissatisfaction or gaps, as these may point to underlying needs	“I think if there was too much kind of input or steering me in one direction that’s not giving authentic service user view” [25]
Expressed Preferences	Preferences indicate needs	“to learn more about the whole person, rather than the ill person they see in hospital or here in the services” [37]
Problem Statements	Problem statements point to opposing needs	“In one case this experience was attributed to a lack of sympathy of the student” [30]
Calls for Improvement	Indications for improvements or changes in existing programmes indicate unmet needs	“There could be more opportunity on placement for learning about service user perspectives and working with self-help/community groups’ [22]

Based on the results of the content analysis, a checklist was developed by YN and refined by EdB. An evidence-based checklist development approach was used, and refinement followed an expert-based approach [33, 38]. First the frequency of the codes was reviewed, then the themes and subthemes were re-interpreted based on the relevance to MHE.

## Results

The inductive content analysis revealed two superordinate themes related to the needs of stakeholders concerning PI in MHE: interpersonal needs and course needs (see Table 5). The needs of patients, educators and students are presented holistically since many themes have relationships with two or all stakeholders in slightly different ways, although reporting the nature of the relationships was beyond the scope of our interpretive strategy. Most themes highlight the needs of patients since most research on PI in MHE is conducted on patients.

**TABLE 5:** Discovered themes of needs of stakeholders concerning PI in MHE (Exploring the Needs of Stakeholders for Successful Patient Involvement in Mental Health Education, Netherlands, 2024).

Superordinate theme	Subordinate theme	Individual codes
Interpersonal Needs	Self-determination	Autonomy [21, 22, 25, 28] Being valued and recognised [21, 25, 27–29] Bring about change [21, 22, 25, 27, 28] Coming to terms with problems [30] Empowering patients [22, 25, 27, 28] Having hope [28] Learning to use own experiences [28]
	Communication and collaboration	Collaboration with mental health institutes [26] Collaborative approach between stakeholders [22, 25–27, 30, 37] Equality between patients and educators [21, 27, 28] Openness to other views [22, 25, 27, 28] Relationship development [21, 23, 24, 30] Trust [22, 28]
	Recognition and Support	Being ensured of employment [27] Companionship [21, 23] Debriefing [21, 23, 27, 28, 30] Emotional support [23, 25, 27, 37] Intellectual support [28, 37] Positive feedback or affirmation [21, 27] Practical support [23, 25, 27] Reduction of us-them thinking [22], [28] Relating to lived experience [22, 26–28] Take into account vulnerability [21, 23, 27, 29]
	Holistic approach	Holistic view on patients [26, 28, 37] Humanising patients [26–29] Positive psychology viewpoint [28, 29] Purposefulness consideration of personal story [22, 25, 27, 30] Self-help or community involvement [22]
Course Needs	Content Needs	Applicable knowledge [26, 27] Balance positive and negative experiences [22, 24, 25, 27, 37] Communicating authentic stories [25, 37] Discussion of therapies [24, 26, 28] Diversity of presented experiences [22, 24, 27] Early exposure to emotional content [27] Focus on emotional intelligence enhancement [27, 28, 37] Incorporate content into assessments [24, 28] Information on mental health journey [27] Learning from uncomfortable teaching [28] More course content [22, 24, 26, 37] Prioritise EBE lessons in the course [22, 24] Promote understanding of mental distress by students [27, 28, 37] Promotion of understanding of broad influence mental health problems [27, 28, 37] Value lived experience above knowledge [26–29]
	Organisational Needs	Address limitations by illness [23] Extend PI content outside of MH education [22, 24, 25, 37] Group discussion with all stakeholders [26–28] Information on mental health journey [27] Make PI course mandatory [24] More integration into the course [22, 22, 27–29] More interaction with students [21, 25, 26, 37] Need for continuous improvement [30] Online education opportunities [27] Practical and organisational resources [22] Prevention of tokenism [21–23, 29] Safe learning spaces to learn together [28] Time to reflect on PI content [25, 27, 37] Involve the right patient [28]
	Teaching Needs	Adapting to student’s knowledge and experiences [25, 37] More interaction with students [21, 25, 26, 37] Avoiding the use of jargon [28] Training in teaching [22, 27, 29] Good communication skills [21, 28] Learning gains from uncomfortable teaching [28]

### Interpersonal Needs

The superordinate theme of *Interpersonal Needs* in the context of PI in MHE encompasses the interpersonal dynamics between patients, educators, and the broader educational system.

The subordinate category of *Self-Determination* depicts the need for autonomy, highlighting the importance of control within the educational contributions of patients. This autonomy extends to decisions regarding the content they share, how they share it, and the impact they hope to make [21, 22, 25, 28]. A quote from a patient from the research of Campbell et al. illustrates this: “I’m waiting to see, that as I say our good faith in terms of how we’re involved and how we’re contributing is recognised.” (p. 343). Patients seek influence in decision-making as well as feelings of empowerment after their involvement in the educational process, desiring a sense of capability and influence. Empowerment enables them to actively contribute to the educational process and contribute to a positive impact on MHE [25].

Another subordinate category of *Interpersonal Needs* is *Communication and Collaboration* between patients and educators (and MHE institutions). Clear communication of shared goals and vision for MHE is often seen as crucial by all stakeholders. This is underscored multiple times by patients’ desire to be seen as equal to educators within the educational process [21, 27, 28]. This equality involves ensuring that patients’ voices, perspectives, and lessons are treated equally to those of educators and MH professionals, which students also acknowledge as necessary. As a student pointed out in Lea et al. [28], “A more equal relationship, not them thinking they are above you” (p. 6). This requires a dialogue that values the input of both parties, and entails acknowledging patients’ expertise and recognising that effective collaboration relies on a reciprocal exchange of trust and understanding [22]. Also, fostering positive relationships between patients, educators, and MH professionals is emphasised. Building trust and rapport creates a conducive environment for effective collaboration, enabling sharing of experiences and knowledge in a respectful environment [21, 23, 24, 30].

A third subordinate category of *Interpersonal Needs* is *Recognition and Support* for PI in MHE, which relates to an often-perceived lack of support for all stakeholders within the educational field. Lived experiences presented by patients may be distressing for all stakeholders, emphasising a need for emotional support opportunities [23, 25, 27, 28, 37]. A proposed way of providing emotional support was to have more time after educational sessions for patients to talk about the lesson and to support each other, as pointed out by students in the research of Horgan [37]. Intellectual support complements this by fostering the intellectual growth of all stakeholders through the lessons taught by patients, ensuring that their contributions are both emotionally validated and intellectually stimulating. Practical support includes assistance in navigating the practical aspects of the course, such as educational systems and software [37]. Stakeholders express a desire for positive feedback and affirmation, depicting the need for explicit recognition of patients’ efforts by both students and educators [21, 27]. Patients’ need for authentic connections with students and educators underscores the importance of valuing personal backgrounds in patient education, fostering empathy and understanding among learners and educators [22, 26–28]. The reduction of us-them thinking centres on breaking down binary distinctions and fostering a sense of shared identity and collaboration. In the context of patient educators, this means dismantling perceived divisions between educators, learners, and individuals with lived experiences [22, 28]. An example of this is the perspective of a patient pointed out by Clarke and Holttum [22]: “It helps develop a different mindset for the trainee (student) - experiencing the other differently but not ‘othering’.” (p. 5).

The final subordinate category of *Interpersonal Needs* is *Holistic Approach,* which includes acknowledging patients as multifaceted individuals, recognising the interconnectedness of their lives beyond MH issues [26–28, 37]. The purposeful consideration of personal stories involves recognising the power of narrative in MHE [22, 25, 27, 28, 30]. In the research by Lea et al. and Meehan et al., there is a recurrent theme of needs, emphasizing a positive psychology viewpoint [28, 29]. This viewpoint underscores strengths, resilience, and wellbeing, and thus departs from deficit-focused approaches to one that highlights the strengths of patients in PI in MHE.

### Course Needs

The superordinate *Course Needs* theme encompasses a commitment to providing a comprehensive, relevant, and empathetic educational experience for students, educators, and patients. The subordinate theme *Content* theme addresses requirements for MHE content, including relevance, balancing positive and negative experiences, authentic storytelling, and therapeutic approaches. When during teaching patients share both successful treatment elements and negative experiences, this can positively impact MHE [22–25, 27, 37]. The quotes of a patient and a student in the research of Kerry et al. illustrate this vividly [27]. Patient: “I hoped that, by discussing good and bad experiences, we could influence future outcomes for clients in a similar position to us.” Student: “I also found it helpful to hear some of their more negative experiences of services, as I have tried to bear those in mind and avoid similar practice on placement.” (p.4). Also, students emphasize the importance of diversity (various life experiences, cultural backgrounds, and cognitive perspectives beyond the neurotypical) in the patient population and lived experiences [22–24, 27]. An example of this is pointed out by a student in Kerry et al.’s research: “it would have been substantially helpful to hear from individuals with lived experience who may not quite fit specific diagnostic criteria, or who may have had alternative reflections on diagnoses.” (p.7) [27]. Furthermore, stakeholders stressed the need for expanded course content and increased integration of patient-led lessons into the curriculum, alongside mandatory PI courses for MH students. This highlights the importance of prioritising PI in MHE beyond current practices. Patients should not merely share their stories as an adjunct to the course; rather, their educational contributions should be seamlessly integrated into the broader curriculum and assessment methods [22–25, 27–29].

The subordinate theme *Organisational Needs* reflects the acknowledgement of specific requirements and considerations needed for successful PI courses at the organisational level. It encompasses how the courses with PI need to be planned, organised, and shaped, such as planning enough time to reflect on PI content and enhancing the integration of the PI content into an overarching course. Prevention of tokenism is a critical aspect of the organisational needs and relates to the subordinate category of *Interpersonal Need* to be recognised and supported as a patient educator. Tokenism occurs when individuals from underrepresented groups are included merely to give the appearance of diversity without truly valuing their contributions [39]. The course has to prevent a tokenistic approach to enable the interpersonal needs discussed above. Thoughtful selection of patients should align their experiences with learning objectives, fostering meaningful contributions and should be selected (by patient educators) based on these requirements [21–23, 29].

The subordinate theme *Teaching Needs* encompasses requirements for impactful learning. This involves adapting teaching methods to suit diverse student experiences and promoting active engagement and collaborative learning. Patients should possess adequate communication skills for teaching [28]. Stakeholders also advocate for PI in the selection of educators to maintain group momentum [21]. Furthermore, training in teaching is pointed out as essential. Acknowledging the value of uncomfortable teaching experiences fosters personal growth, encouraging the navigation of challenging situations and reconsidering assumptions [28].

### A Comprehensive Checklist for Implementing PI in MHE

Based on the findings described above (also see Table 5), we developed a checklist (see Supplementary Material) for potential stakeholders to incorporate more frequent and more active PI in MHE. This checklist can serve as an evidence-based starting point for educators and curriculum developers in MHE to expand and assess PI in MHE. The checklist is compiled with the two main themes, and their subthemes that emerged from the review as described above: *Interpersonal Needs* consisting of *Self-determination, Communication and Collaboration, Recognition and Support*, and *Holistic approach*, and *Course Needs* consisting of *Content, Organisational*, and *Teaching*. For each theme, a comprehensive list of questions is provided that ensures a thorough check of all aspects of implementing or augmenting PI in MHE. This is the first comprehensive checklist for implementing PI in MHE based on a comprehensive literature review.

## Discussion

This scoping review aimed to comprehensively map the existing literature on PI in MHE and analyse and identify the needs of MH educators, students, and patients with lived experiences of MH challenges and develop a checklist for successful implementation of PI in MHE. This scoping review contributes to the field by providing a preliminary map of evidence and providing a checklist for stakeholders aiming to incorporate PI in their MHE programmes.

### Implications for MHE

The primary research questions, focusing on the needs of stakeholders in MHE, were addressed by an inductive content analysis and revealed two superordinate themes. These are *Interpersonal Needs,* consisting of the subordinate themes of *Self-determination, Communication and Collaboration, Recognition and Support* and a *Holistic Approach, and Course Needs*, consisting of *Content Needs*, *Organisational Needs* and *Teaching Needs.* Based on these themes, our checklist for successful PI in (MHE) emphasises patient autonomy, collaboration, and communication among all stakeholders with mutual respect and equality. Providing emotional and intellectual support for all parties, along with practical assistance for patients, is crucial. Additionally, adopting a positive psychology perspective that portrays patients as multidimensional individuals beyond their symptoms (see e.g., [40]) is essential for fostering positive interpersonal relations. In terms of the course, guidelines should focus on creating balanced and inclusive content that integrates patient stories effectively. Patients should also receive training for teaching and play an active role in selecting suitable candidates for MHE.

### Implications for Research

The literature on PI in MHE reveals several gaps that need further research. There is a lack of standardised methods in research on PI in MHE, hindering the reliability of findings in this field. Standardised methods and consistent procedures could fill this gap.

The contextual variations present within different programmes and institutions in MHE are not adequately researched and thus present one of the literature gaps. For example, MH nursing and psychiatry involve medically schooled students and educators. Both disciplines fall between the social sciences and medical sciences, taking a different stance than clinical psychology students and educators who are social scientists [41]. Consequently, it becomes crucial for research efforts to not only acknowledge these contextual nuances but also actively seek to identify and address the specific challenges and opportunities they present. For instance, a PI intervention that proves successful in one psychiatric or MH training program may encounter obstacles or require modifications when implemented in a clinical psychology curriculum. Understanding these differences is necessary for tailoring PI strategies to suit the needs of each educational context, ultimately heightening the effectiveness of PI initiatives across various fields within MHE. Important criteria for developing these guidelines could be e.g., the relevance of the course content and assessment criteria to the specific educational needs per field. Our checklist is a preliminary attempt, yet more comprehensive guidelines need further research. Interpersonal needs criteria that may be researched are the possible differences in communication and collaboration between stakeholders in the field. Research on the differences between stakeholder needs in various fields of MH could include a programme-specific needs assessment and a qualitative exploration of stakeholder needs. After this, future research could explore strategies to adapt PI initiatives to meet the specific needs of different MHE programmes to tackle programme-specific challenges.

In this scoping review, PI mainly centred on presenting patients’ own lived experiences. Patients at higher levels of involvement (level 5 or 6 according to Towle et al.) are expected to express less desire for further involvement, empowerment, autonomy, and collaborative approaches, as these aspects are fundamental to their active educational role. Further research is needed to explore the needs of patients at different levels of Towle et al.’s taxonomy. Nonetheless, we recognise that since cutoff of our search, more studies with focus on PI in MHE have been published [42], including a qualitative systematic review, albeit with a different focus [43]. We acknowledge that we are situated in a dynamic research field and that there is the need for regular and rapid reviews to synthesize the knowledge produced in this emerging arena.

### Limitations of the Study

One limitation of the current study is the subjective nature of the inductive content analysis and checklist development, wherein implicit references were extracted to uncover stakeholders’ underlying needs. The initial review and data analysis process relied for a large part on the perspective of MK who was inherently involved in PI in MHE as a clinical psychology intern at the time of data analysis. Additionally, supervision and checklist development were undertaken by YN and EdB, clinical psychology practitioners, researchers and educators, who are themselves stakeholders. This positionality underscores the potential for bias in extracting and synthesising information, as individual perspectives may influence the identification and interpretation of stakeholders’ needs [44]. To address this, future research could validate these findings through qualitative studies guided by the same research question, involving multiple researchers [45].

The absence of a quality assessment of the included studies, due to scoping nature of the review, constitutes another limitation. This absence of methodological evaluation may have led to the inclusion of methodologically flawed studies, potentially impacting the overall reliability of the findings. This limitation underscores the need for caution in generalising the identified needs, as the quality and validity of the included studies were not systematically appraised, and furthermore underscores the need for studies in this field with a sound methodological approach.

Furthermore, the analytical strategy employed in this study did not permit interpreting the interconnectedness of relationship between the needs of the different stakeholders. The relational nature of education, especially professional education, implies that the needs of one stakeholder is inherently connected to the needs of another. Additionally, due to the power differentials within the hierarchical structure of MHE, one stakeholder may be responsible for meeting the needs of the other. A full relational analysis was beyond the scope of this study. Future research studies could employ analytical approaches such as Situational Analysis to allow for a more comprehensive relational and positional analysis [46].

Lastly, there is a possibility that publications were not captured although efforts were made to include a diverse range of databases. The inclusion criteria also focused on English-language studies, potentially leading to the exclusion of relevant non-English literature that could provide valuable cross-cultural insights. This is likely, considering the high inclusion of UK, Ireland, and Australia studies. The overrepresentation of research from the Happell et al. group suggests potential publication bias. To enhance understanding, future research should include diverse patient perspectives from additional countries and cultures. Comparative studies examining caregivers’ viewpoints can also contribute to a comprehensive understanding of PI [47].

### Conclusion

In conclusion, this scoping review provides a comprehensive overview of the existing literature on PI in MHE and identifies the needs of stakeholders, including students, MH professionals, and patients with lived experiences for successful PI implementation in MHE. Through an inductive content analysis, two overarching themes emerged: *Interpersonal Needs* and *Course Needs*, each comprising several subordinate themes. A checklist was constructed based on the content analysis findings. These offer valuable insights and practical tools for curriculum developers, educators, policymakers, and researchers, laying the groundwork for evidence-based guidelines to enhance PI in MHE. Addressing the identified gaps, such as standardising research methods, understanding contextual variations across MHE programs, and including diverse patient perspectives, presents opportunities for future research and practice in this area. Despite limitations, including the subjective nature of the analysis and potential publication bias, this study’s comprehensive approach contributes to advancing our understanding of PI in MHE and underscores the importance of collaborative efforts to promote patient-centred education and practice in mental health.
