# Supplementary material for: Exploring the Needs of Stakeholders For Successful Patient Involvement in Mental Health Education
Source: Public Health Rev. 2025 Mar 12;46:1608124. doi: 10.3389/phrs.2025.1608124 (PMC11936750; doi:10.3389/phrs.2025.1608124)
Supplement: Supplementary file 1 [file DataSheet1.pdf]

Supplementary Material. Checklist for Needs of Stakeholders Concerning Patient Involvement (PI) in Mental Health Education (MHE)

© Marlin Klarenbeek, Ed de Bruin, Yudit Namer

Interpersonal Needs

Self-determination:

- Are the patient educators (PEs) given autonomy in their teaching?
- Do PEs have control over the decisions regarding the content they share, how they share it, and the impact they hope to make?
- Are the PEs valued and recognised within the MHE program?
- Are PEs given opportunities to bring about change within the MHE program?

Communication & Collaboration:

- Is there a collaboration with mental health institutes?
- Is there an inclusive MHE vision that reflects the diversity of perspectives within the collaboration?
- Is there a clear communication of shared goals and vision for MHE among all stakeholders?
- Is equality between PEs and non-patient educators ensured?
- Are there opportunities for relationship development (based on trust and rapport) among all stakeholders?

Recognition & Support:

- Are PEs ensured of employment?
- Is there equitable and sufficient remuneration for PEs?
- Are there emotional support mechanisms for PEs (examples may include debriefing, remunerated time to process the course material)?
- Are there intellectual support mechanisms for PEs (examples may include teachers' training courses)?
- Are there practical support mechanisms for PEs (examples may include assistance in navigating the practical aspects of the course, such as educational systems and software)?
- Are potential vulnerabilities of PEs taken into account?

Holistic Approach:

- Are PEs seen as full, multifaceted individuals?
- Is there a positive psychological lens in the MHE vision?

Course Needs

Content Needs:

- Is the knowledge conveyed applicable for students?
- Does the course content balance positive and negative experiences of PEs?
- Does the course content include discussion of therapies from the PEs' perspective?
- Is there early exposure to PE content early on in the course?
- Does the course content focus on enhancement of emotional skills?
- Is the course content incorporated into assessment?
- Does the course content prioritize lived experience?

Organisational Needs:

- Is there a good fit between the PE and the course taught?
- Is PI course mandatory or encouraged?
- Is there sufficient interaction of PEs with students?
- Are there measures in place to avoid tokenism?
- Are safe learning spaces facilitate?
- Do all stakeholders have sufficient time to reflect on PI content?

Teaching Needs:

- Is the PI content adapted to students' knowledge and experiences?
- Are the PEs given training in teaching and communication skills?
- Are there opportunities to address discomfort?
